# Axial muscle-fibre orientation in developing larval zebrafish

**DOI:** 10.1242/jeb.250905

**Published:** 2026-01-02

**Authors:** Noraly M. M. E. van Meer, Johan L. van Leeuwen, Martin J. Lankheet

**Affiliations:** Experimental Zoology Group, Wageningen University, 6708 WD Wageningen, The Netherlands

**Keywords:** Muscle development, Fast white muscle fibres, *Danio rerio*

## Abstract

The fast axial muscles of larval bony fish power rapid escape responses, crucial for survival under high predation pressure. In adult and juvenile teleosts, these muscles follow a pseudo-helical pattern, while those of larval zebrafish (*Danio rerio*) exhibit a helical arrangement. However, its developmental time line remains unclear. We analysed muscle-fibre orientation in genetically modified zebrafish from 2 to 13 days post-fertilization (dpf) using 3D fluorescence confocal microscopy. Fibre angles were quantified relative to the notochord, and a model of circular, concentric helices was fitted to assess helical trajectories across development. Our results show that a helical pattern is already present at 2 dpf, tapering towards the tail. The pattern remains stable over the first 11 days post-hatching, with decreasing projection angle variation toward the tail, particularly in younger larvae. Across developmental stages, helix centres align at corresponding normalized positions along the notochord. This study highlights the early presence of a helical fibre arrangement in larval fish, with no evidence of pseudo-helical deviations up to 13 dpf.

## Teaser

**Summary:** A helical muscle-fibre arrangement is present in larval zebrafish immediately after hatching, with no major changes during the following 11 days, and can contribute to effective swimming during early life.

## INTRODUCTION

In most teleost fish, white muscle fibres make up the bulk of the axial muscles, enabling fast, powerful escape responses, quick turning manoeuvres and high-speed cyclic swimming bursts (Jayne and Lauder, 1994; Altringham and Ellerby, 1999). These fibres also contribute to suction feeding (Thys, 1997; Carroll and Wainwright, 2006; Camp et al., 2015, 2018, 2020; Jimenez and Brainerd, 2020; Li et al., 2022; Jimenez and Camp, 2023), biting (Alfaro et al., 2001) and pharyngeal chewing (Sibbing et al., 1986). The axial muscle fibres are segmented into myomeres, interconnected by folded connective tissue sheets called myosepts.

The arrangement of axial muscle fibres has been described as helical, with pitch angle increasing outward from the centre, where fibres run nearly parallel to the body axis (Alexander, 1969; Gemballa and Vogel, 2002). However, deviations from this pattern have been observed in juvenile and adult fish creating a ‘pseudo-helical pattern’. This pattern differs from a perfect helix for fibres close to the median plane, where they show larger angles with the median plane (higher azimuth values) at the level of the helix centre than predicted for a helix (Mos and Van der Stelt, 1982; van Leeuwen et al., 2008). This arrangement is thought to minimize variation in muscle strain across a transverse slab during lateral bending, facilitated by shear deformation (Mos and Van der Stelt, 1982; van Leeuwen et al., 2008).

In larval fish, rapid muscle development is critical for survival (Osse, 1989), as predation pressure is extremely high; only 0.1% of larval fish reach the juvenile stage (Bailey and Houde, 1989; Fuiman and Cowan, 2003; China and Holzman, 2014). Larvae begin moving inside the egg and can swim immediately after hatching. Unlike adults, larvae possess only two muscle fibre types: a thin layer of slow red fibres under the skin and fast white fibres that comprise most of the muscle volume. When muscle cells form, they progress through several precursor stages. At the 14-somite stage, the uni-nuclear myoblasts that are the precursors for fast muscle cells align and fuse to form poly-nuclear myotubes that are oriented longitudinally in the body (van Raamsdonk et al., 1974). While the initial fibre arrangement appears longitudinal, conflicting studies describe its transition toward a helical or pseudo-helical configuration.

van Raamsdonk et al. (1974) first described a ‘helicoid’ fibre pattern at 6–7 days post-fertilization (dpf), while van Leeuwen et al. (2008) observed deviations from a strictly longitudinal pattern as early as 1 dpf, becoming more pronounced by 3 dpf. In contrast, Dou et al. (2008) reported that most muscle fibres at 5–7 dpf remained longitudinal. More recently, van Meer et al. (2025) quantified fibre angles along the full axial muscle length in 4 dpf fish, finding a helical, not pseudo-helical, pattern with decreasing pitch angles toward the tail.

With strikingly different reported results concerning the timing of the helical muscle-fibre arrangement development, it remains unclear when the muscle-fibre trajectories become helical or pseudo-helical, and how much the muscle-fibre arrangement changes from day to day as larval fish develop. So far, little information about the muscle-fibre arrangement along the entire axial muscles during larval development is available, despite changing functional demands over the anteroposterior axis and with age. Here, we investigated the fast axial muscle-fibre arrangements across the entire axial musculature of zebrafish larvae from 2 to 13 dpf, using the methodology of van Meer et al. (2025). We fitted a helix model to the quantified muscle-fibre orientation to investigate (1) to what extent the muscle-fibre arrangement differs from a circular helical pattern, (2) the position of the helix centres, and (3) changes in helix pitch along the body. Previously, we showed that the helical muscle-fibre arrangement was already present in zebrafish larvae at 4 dpf. Here, we examined whether this arrangement already exists when the fish hatch at 2 dpf and how this arrangement evolves with development. Given its potential role in minimizing strain variation (van Leeuwen et al., 2008), which may provide an immediate functional advantage during their typical high tail-amplitude swimming motions (Müller and Van Leeuwen, 2004; van Leeuwen et al., 2015), early establishment of a helical fibre pattern may provide a functional advantage in predator evasion, a key factor in larval survival.

List of symbols and abbreviations

*c*: a constant for the slope in the pitch of the helix from the helix centre outward on the transverse plane
dpf: days post-fertilization
ℓ_p_: the pitch of the helix
*R*: the radial distance to the helix centre
*x*, *y*, *z*: the longitudinal, dorsoventral and mediolateral coordinates of a point on a helix, respectively
*x*_c_, *y*_c_, *z*_c_: the longitudinal, dorsoventral and mediolateral coordinates of the helix centre, respectively
*v*: the unit direction vector of the helix model
*v*_t_: the length of *v* in the transverse plane
*v_y_*: the dorsoventral component of *v*_t_
*v_z_*: the mediolateral component of *v*_t_
α: azimuth: the frontal projection angle of a muscle fibre
α_m_: the frontal projection angle of the helix model
β: elevation: the angle between a fibre and the frontal plane
ζ: the angle around the cross-sectional circle of the helix
θ: the angle a muscle fibre makes relative to the fish's straightened notochord
τ: pitch angle of the helix
ϕ: the sagittal projection angle of a muscle fibre
ϕ_m_: the sagittal projection angle of the helix model

## MATERIALS AND METHODS

Muscle-fibre orientation of larval zebrafish, *Danio rerio* (F. Hamilton 1822), of 2–13 dpf was quantified from high-resolution confocal fluorescence microscopy scans. This study used the methods described in detail by van Meer et al. (2025), following the same procedures for data collection and analysis. These methods are summarized below with modifications specified where necessary. Specimens older than 5 dpf were obtained from surplus animals raised as part of normal stock maintenance and were euthanized according to stock maintenance protocols, after which they were available for imaging. Animal care and experiments were approved by the Wageningen University animal welfare officer.

### Animals, embedding and imaging

**Fig. 1.**
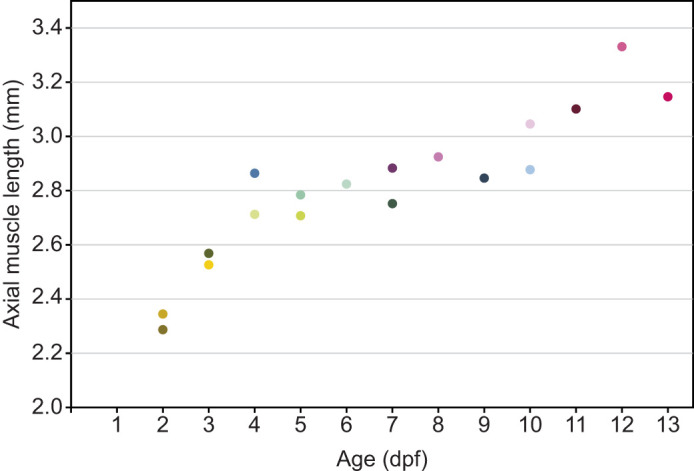
**Axial muscle length of the larval zebrafish per age.** The length of GFP-labelled axial muscle tissue is shown against age in days post-fertilization (dpf). The colour labels for individual fish match those shown in the plots of Figs 7 and 8.

For visualizing the 3D arrangement of axial muscle fibres, we used a genetically modified zebrafish line (*Danio rerio*; casper mylz2:GFP) with fluorescent fast muscle fibres. Green fluorescent protein (GFP) was expressed in fast skeletal muscle fibres, using the specific promotor for myosin light polypeptide 2 (mylz2) (Ju et al., 2003). Expression in a pigmentless, albino line rendered fluorescence visible throughout the axial musculature. The expression level and fluorescence intensity varied randomly among fibres, allowing for individual fibre segmentations. The larvae were kept in a 1 litre tank in Danieau's solution (Godinho, 2011), at 28°C and fed artemia twice a day after 5 dpf. Larval fish of 2–5 dpf were anaesthetized as described previously (van Meer et al., 2025), and fish of 6–13 dpf were euthanized with MS-222 (MS222: 20 mg 100 ml^−1^, bicarbonate: 40 mg 100 ml^−1^) to meet animal-experiment regulations. To fix their position for confocal imaging, fish were embedded in 1.5% low melting point (type VII) agarose (UltraPure Agarose, Invitrogen) on an embedding slide and oriented as close to a straight and horizontal position as possible to optimally align their central axis for 3D scanning. The agar was then solidified on an ice pack in about 10 s, and the fish in agar was then covered with a coverslip to prevent dehydration. Scans at 25× magnification (HC FLUOTAR L 25 × 0.95 W VISIR, Leica Microsystems) covered their entire length and at least half the width. Our methods yielded scans with sufficient contrast to segment individual fibres. Muscle depth was not a limiting factor in resolving the data, as the fluorescence of the muscles was very bright, and further analysis separated individual fibres based on their intensity differences with their direct neighbours. For additional scans of a 1 dpf individual, we hatched an individual with fine-tipped forceps and prepared it for scanning as described above. Because of the larva's high body curvature around its relatively large yolk sac, we scanned it at three longitudinal positions with a ×40 magnification (HC PL APO CS2 40×/1.30 OIL objective). This individual was not included in quantitative analyses, but is described qualitatively in the Results section. In analysing the data, we assumed bilateral symmetry, and, where necessary, fibre data were mirrored to the right side of the fish to compare between individuals. Because accurate measurements of standard length were impossible from the scans, we used the length of fluorescent axial muscles as a proxy for the length of the fish, assuming isometric growth. The axial muscle length was calculated as the distance between the anterior- and posterior-most axial muscle tissue. We scanned at least two fish per age group. The number of fibres included in the analysis depended on scan quality, which varied per fish. The 4 dpf individuals were reused from van Meer et al. (2025). Fig. 1 presents the axial muscle lengths for all fish. To take into account the variation in the length–age relationship, we studied developmental changes both as a function of age and as a function of fish size. To compare results between animals, we used the colour labels shown in Fig. 1.

### Image processing and analysis

**Fig. 2.**
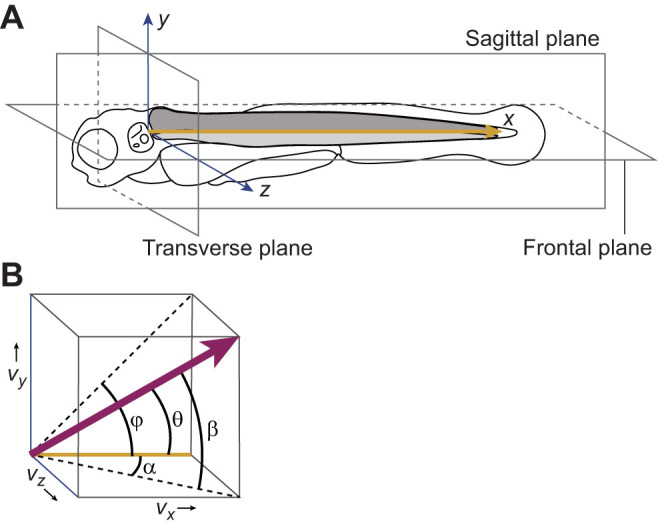
**Schematic overview of the planes, axes, and angles.** (A) Coordinates are defined relative to the centre line *x* of the notochord, with *x* increasing in the posterior direction, *z* in the lateral direction and *y* in the dorsal direction. The origin in the longitudinal direction is aligned to the anterior-most muscle attachment to the cranium. Because of the natural bending of the anterior-most part of the notochord, the centre of the notochord deviates slightly from the central axis for lateral body curvatures. (B) Definitions of muscle-fibre orientations of a vector (*v_x_*,*v_y_*,*v_z_*,). α, frontal projection angle (also called azimuth); ϕ, sagittal projection angle; β, elevation; θ, angle with the centre line of the notochord. Adapted from van Meer et al. (2025).

Raw 3D scans were deconvolved with Leica's Lightning Process (Leica Microsystems) and scans were processed with custom-written Python programs. The images were filtered to improve contrast and remove noise before segmentation. A watershed algorithm was used to segment the images and the segmented objects were filtered on size to select for individual muscle fibres. For each segmented fibre, we measured its size, centroid and orientation. The location and orientation of a fibre were defined relative to the centreline of the notochord, using an orthogonal projection. This corresponds to a digitally straightened notochord, defining a coordinate system where the *x*-coordinate increases from anterior to posterior along the notochord (Fig. 2); *z* represents the mediolateral axis and *y* represents the dorsoventral axis, both with their origin at the centre of the notochord. In digitally straightening the notochord and constructing the coordinate system, we corrected for misalignments in fish embedding, body curvature and torsion.

Using the centreline of the notochord as a reference, we created a normalized coordinate system that allowed us to compare muscle architecture between individuals and across developmental stages. This coordinate system does not directly match the actual axes for lateral body curvature; especially anteriorly, the angles may deviate substantially, as a result of the flexure of the notochord. This needs to be considered when predicting, for example, the effect of fibre orientation on strain, which in turn depends on estimates of actual body undulation.

Muscle-fibre orientation was initially calculated as unit vectors **v**=(*v_x_*_,_*v_y_*_,_*v_z_*), with *v_x_* pointing posteriorly. For comparisons and visualizations, we determined two angles for each segmented muscle fibre (Fig. 2B): (1) the angle between the projection of **v** onto a frontal plane and the *x*-axis [frontal projection angle α; α=arctan(*v_z_*/*v_x_*)], and (2) the angle between the projection of **v** onto a sagittal plane and the *x*-axis [sagittal projection angle ϕ; ϕ=arctan(*v_y_*/*v_x_*)]. Thus, we defined α and ϕ equivalently in orthogonal planes. The former is identical to the previously defined azimuth, whereas the latter differs from the previously defined elevation (angle β, see Fig. 2B) (van Leeuwen et al., 2008; van Meer et al., 2025). Frontal projection angles are defined positive for *v_z_*>0 (pointing laterally). Sagittal projection angles are defined positive for positive *v_y_* (pointing dorsally). The combination of α and ϕ fully defines the orientation of a muscle fibre. To quantify the fibre orientation relative to the longitudinal axis, we also computed the angle θ between **v** and the central axis of the notochord {θ=arctan[√(*v_z_*^2^+*v_y_*^2^)/*v_x_*]; θ≥0}. After applying the initial criteria to filter out erroneous fibre segmentations that resulted from the merging of multiple fibres during segmentation, or from incomplete segmentations, there were still some obvious outliers. Results were deemed outliers, and unlikely to represent individual fibres, if α, β or θ was larger than 45 deg. Any remaining fibres with unlikely high deviations from surrounding fibres were manually inspected and removed if the segmentation did not represent a single, complete muscle fibre.

### Data analysis: fitting a helix model

The helical pattern as previously described for fibre arrangements in axial muscles (Alexander, 1969; Gemballa and Vogel, 2002; van der Stelt, 1968; Mos and Van der Stelt, 1982; van Leeuwen et al., 2008) consists of nested helices (or pseudo-helices), with a centre where fibres run in the longitudinal direction and with the pitch angle of the helix increasing as a function of distance to this centre. With an increasing pitch angle, the frontal and sagittal projection angles (α and ϕ) will also radially increase.

### Model equations

The location of a point on a helix is given by (*x*,*R*,ζ), where *x* is the longitudinal coordinate, *R* is the radial distance to the helix centre and ζ is the angle around the cross-sectional circle of the helix (Fig. S1). For a fibre at location (*y*,*z*) and helix centre (*y*_c_, *z*_c_), *R* is given by:
(1)

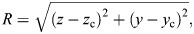
and ζ is computed as:
(2)

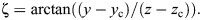
We now need to fit the projection angles of the helix model (α_m_,ϕ_m_) to the measured projection angles. For the helix model, we can calculate the projection angles from the unit direction vector and the pitch angle of the helix. Let the unit direction vector be **v**=(*v_x_*_,_*v_y_*_,_*v_z_*). The projected length of **v** in the transverse plane is:
(3)

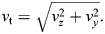
For the components of *v_y_* and *v_z_*, we derive:
(4)

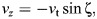

(5)

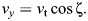


The minus sign for *v_z_* aligns the values with the definitions of directions in the different quadrants (leftward as minus in the first quadrant). For the pitch angle of the helix τ, it holds:
(6)

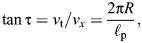
where ℓ_p_ is the pitch of the helix. And for the modelled projection angles, it holds:
(7)

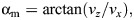

(8)

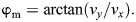


From Eqns 4, 6 and 7, it follows:
(9)

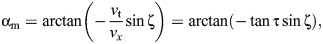
and similarly from Eqns 5, 8 and 6:
(10)

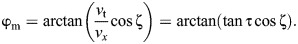
As a first approximation, we assumed a constant pitch ℓ_p_ for different distances to the helix centre. This requires that:
(11)

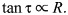
We can then rewrite Eqns 9 and 10 as:
(12)

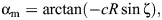

(13)

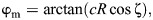
where *c* is a constant that we call the slope. From Eqn 6, the pitch estimate of the model fit can be derived:
(14)

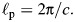
To fit the model to the full epaxial and hypaxial datasets for a fish, we need to incorporate possible variations of *z_c_*, *y_c_* and *c* along the notochord. Hence, *z_c_*, *y_c_* and *c* are functions of *x*: *z_c_*=*z_c_*(*x*), *y_c_*=*y_c_*(*x*), *c*=*c*(*x*). For *z_c_* and *y_c_*, we modelled the variations by a fourth-order polynomial, and for *c* by a third-order polynomial:
(15)

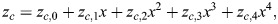

(16)

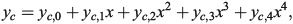

(17)

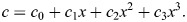
The model was fitted separately for the epaxial and hypaxial muscles, using the full datasets for each individual fish, with the *curvefit* procedure in the *SciPy* module (virtanen et al., 2020) in Python. Confidence intervals (95%) for *z_c_*, *y_c_*, ℓ_p_ at each location along the notochord were calculated using the covariance matrix from the fit procedure, taking contributions from the different polynomial coefficients into account (error propagation). Fits were performed using normalized length values, with the observed anterior-most fluorescent muscles as *x*=0 and the posterior-most values as *x*=1. Our coordinate system is defined relative to the centre of the notochord. Depending on notochord flexure in the anterior part, this may deviate from the actual orientation relative to a forward swimming direction. For analysing the properties of the helical structure, we assumed that the notochord reference is most appropriate. In determining how the specified structures affect swimming performance, the actual location and orientation relative to swimming movements need to be taken into account.

### Statistics

To investigate whether helix pitch differed among fish larvae, we extracted helix pitch estimates and their corresponding 95% confidence intervals from the helix model at five standardized locations along the body axis (30%, 40%, 50%, 60% and 70%). To account for variation in measurement precision, the 95% confidence intervals were converted to standard errors and incorporated as inverse-variance weights in the analysis. Helix pitch was then modelled as a function of axial muscle length and body location using a linear mixed-effects model with a random intercept for each fish. The model was implemented in the nlme package in R (v4.4.3). Model assumptions were evaluated by inspection of residual plots to verify homoscedasticity and normality.

### AI use

ChatGPT (OpenAI 2023) was used as a tool in text improvement and as initial code generator for the model. The authors subsequently reviewed and edited the content as necessary before implementation and take full responsibility for the publication’s final content.

## RESULTS

Eighteen larval fish, ranging in age from 2 to 13 dpf provided good coverage of measurements across the entire axial muscle length. In total, 9751 muscle fibres were quantified, amounting to an average of 542 fibres per fish. Axial muscle length increased by about 50% from ∼2.3 to 3.1 mm for the age range of 2–13 dpf (Fig. 1). Length, however, did not fully correlate with age. Because fish length is in many cases a better indicator for developmental stage than age (Parichy et al., 2009; voesenek et al., 2018), we also used axial muscle length as a proxy for ontogenetic stage. Unless stated otherwise, our conclusions did not depend on one or the other quantification of development.

### Muscle-fibre orientation

**Fig. 3.**
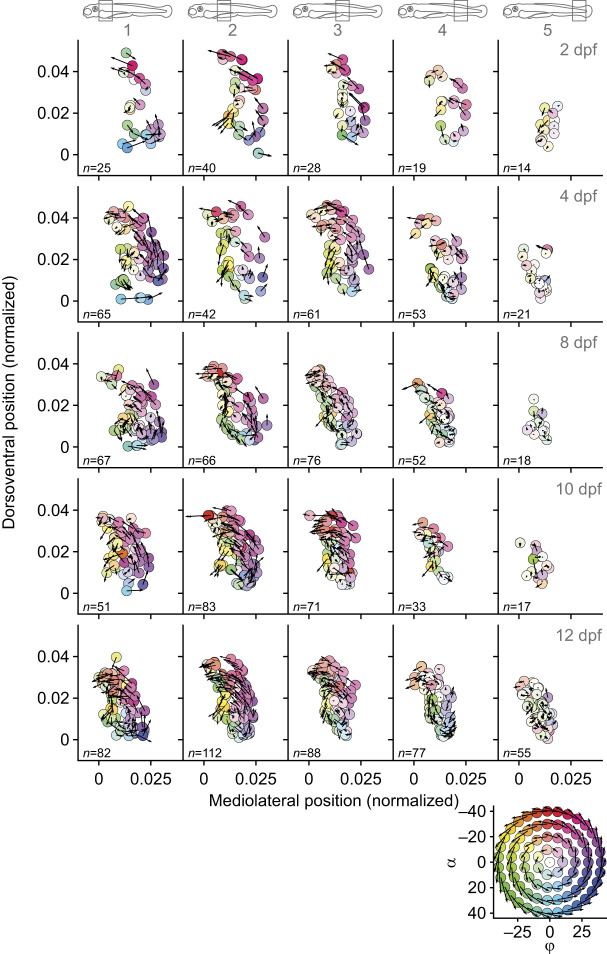
**Muscle-fibre orientation in the epaxial muscles for five bins along the notochord (see icons at the top) for five representative ages.** Each row of panels represents measurements for a single larval fish, with the number of fibres shown in the bottom left corner of each panel. The centre of the notochord is defined as (*y*,*z*)=(0,0). Fibre coordinates and orientations are shown as a combination of a vector plot and a colour-coded scatter plot. Fibre coordinates were normalized so that the full length of the axial muscles is 1 and the other axes are scaled accordingly. The arrows represent (*v_z_*,*v_y_*) components of the unit-direction vectors in the transverse plane (i.e. perpendicular to the centreline of the notochord). The same scale factor was used for all vectors. Short arrows at or near the centres of the hypothesized helical patterns indicate small deviations from the longitudinal direction. In these views, frontal projection angles correspond to deviations from vertical, and sagittal projection angles to deviations from horizontal (Fig. 2). The combination of frontal and sagittal projection angles defines the hue for the scatter plot, as indicated by the coloured circles in the inset (bottom right). The saturation corresponds to the combined magnitude of projection angles, (α^2^+ϕ^2^)^1/2^. The inset shows the pattern of angles for a perfect helix, with projection angles varying sinusoidally along circles, and increasing linearly with distance from the centre. To illustrate the change in orientation along the length of the fish, data are shown in five bins from anterior to posterior, each covering 20% of the total axial muscle length.

**Fig. 4.**
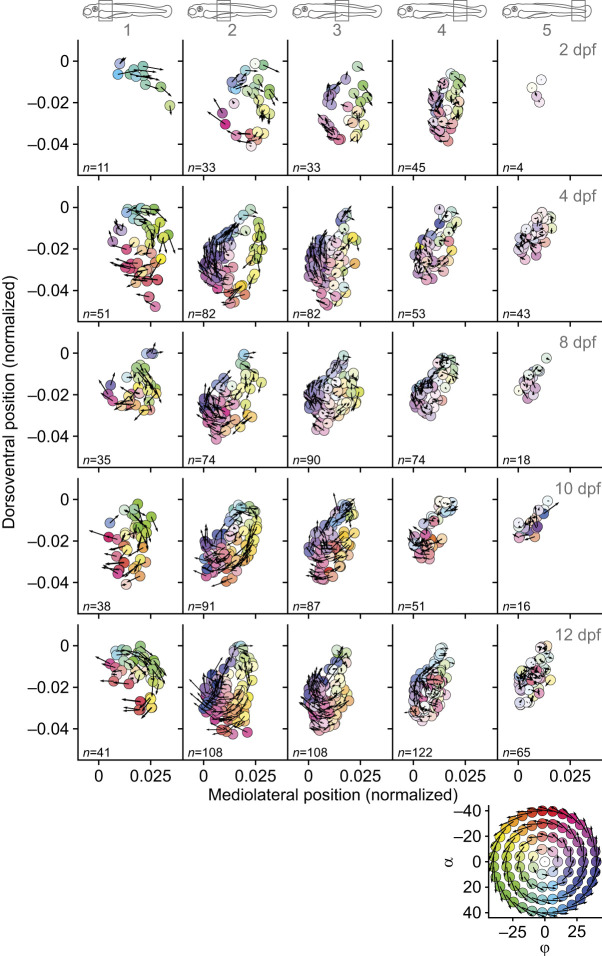
**Muscle-fibre orientation in the hypaxial muscles for five bins along the notochord (see icons at the top) for five representative ages.** Results are presented in the same way as in Fig. 3.

Figs 3 and 4 show muscle-fibre orientation in five bins (slabs) along the body for five representative fish of 2, 4, 8, 10 and 12 dpf. For the epaxial part of the axial muscles (Fig. 3), the fibre orientations reflect the structure visible in the inset, where vector components vary with the cosine and sine along concentric circles. The highest positive frontal projection angles (α) are found near the horizontal septum (*y*=0) and the most negative values are at the most dorsal positions. Variations in sagittal projection angles (ϕ) are rotated by 90 deg relative to the frontal projection angles. Sagittal projection angles are maximal at the lateral-most locations and minimal near the median plane (*z*=0). The magnitudes of projection angles display the same radial pattern as in the inset, reflecting an increase from a central location, where angles are minimal. These patterns were previously described for 4 dpf fish (van Meer et al., 2025); here, we see qualitatively similar patterns for all ages. The magnitude of projection angles clearly depends on the anteroposterior location along the fish. The highest angles are found anteriorly, and in all cases the magnitude diminishes towards the tail. In the posterior-most bin, the fibres are oriented nearly parallel to the notochord. These patterns resemble a structure of concentric helices with a centre where fibres run approximately in the longitudinal direction, and with increasing pitch angle for larger distances to the centre. However, there is considerable variation relative to the perfectly regular concentric helices as shown in the inset. For the hypaxial part of the axial muscles (Fig. 4), the structure is very similar, but the helix changes angle in exactly the opposite direction. This corresponds to mirroring the structure relative to the horizontal septum (*y*=0). The ventral-most fibres have the most negative frontal projection angles and the fibres near the horizontal septum have high positive projection angles. Similarly, the signs of sagittal projection angles are reversed compared with the epaxial musculature. Hypaxial and epaxial muscles also differ in the space that they occupy: epaxially, they wrap around the space occupied by notochord and the neural arch, whereas hypaxially, they wrap around the notochord, and in the anterior part they arch slightly over the abdominal cavity.

**Fig. 5.**
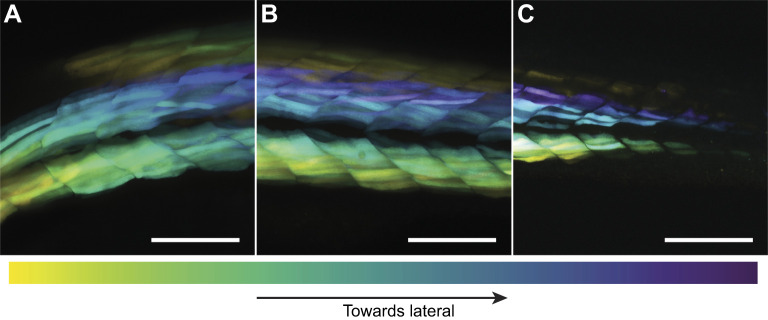
**Semi-lateral 3D scans of a 1 dpf zebrafish larva.** Data represent colour-coded maximum projection of the raw 3D data of a 1 dpf larval fish. Colours correspond to the raw *z*-coordinate, in a gradient from yellow to dark blue from medial to lateral, as indicated by the colour scale. (A) Anterior myomeres, (B) myomeres around the anus and (C) posterior myomeres. Scale bars: 0.1 mm.

Scans of the 1 dpf larva (Fig. 5) show that fibre orientation already deviates substantially from the longitudinal direction before hatching. It is most clearly visible in Fig. 5B, where orientations of lateral epaxial fibres (purple) deviate from those of the medial fibres (light blue). The observed orientations are consistent with a helical pattern similar to that found for the next developmental stages. Unfortunately, because of the high body curvature, we were unable to accurately quantify the angles of the muscle fibres in this young specimen. Nonetheless, the data qualitatively confirm the presence of a helical pattern at this pre-hatching stage.

### Helix model

**Fig. 6.**
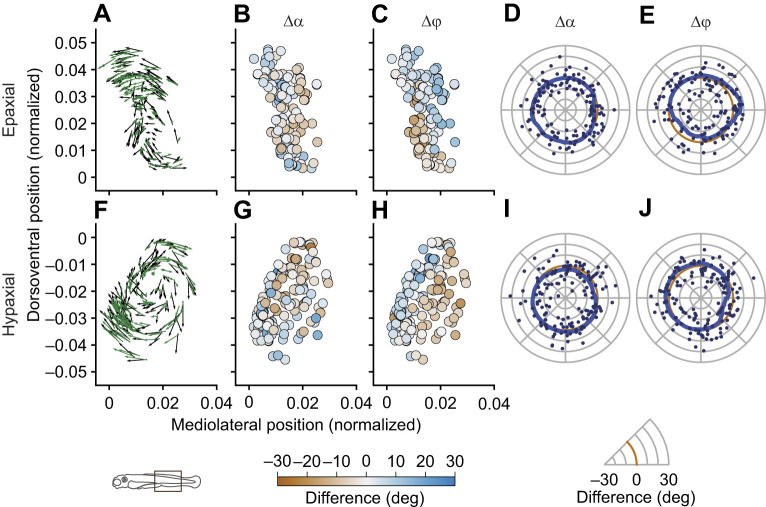
**Representative example of a helix fit with errors for the epaxial and hypaxial muscles in the top and bottom row, respectively.** This example is from a 3 dpf fish and contains the muscle portion between 0.4 and 0.8 (see icon at the bottom) on the normalized anteroposterior axis. (A,F) Vector plots of the original data on the transverse plane in black, and the fitted data in green. The arrow direction represents the orientation of the muscle fibre, and arrow length represents the vector magnitude. The longer the arrow, the larger the angle with the fish's longitudinal axis. (B,G) Difference between the frontal projection angles of the original and fitted data. (C,H) Difference between the sagittal projection angles of the original and fitted data. These differences, or errors, are colour coded, as indicated by the colour scale. (D,E,I,J) These same errors, now shown as a function of the direction (angle ζ) relative to the centre of the helical structure. The zero level is indicated by the orange line. Symbols represent individual fibres, and the solid blue line shows a moving average with a 90 deg window size. This relatively large window size effectively removes noise from the data, while maintaining major asymmetries. Systematic, direction-related errors show up as a difference between the blue and orange lines. (D,I) Polar plot with errors in frontal projection angle (α) as a function of direction relative to the helix centre. (E,J) Polar plot with errors in sagittal projection angle (ϕ) as a function of direction relative to the helix centre.

To quantify the observed muscle-fibre arrangement, we fitted a helix model to the data. First, we examined how well the helix model describes the observed patterns and to what extent systematic differences relative to the concentric helix model are present. In Fig. 6, we show a representative example of the helix fitted in one of the 3 dpf fish. For this example, we selected data for the middle section of the fish only (0.4<*x*<0.8).

**Fig. 7.**
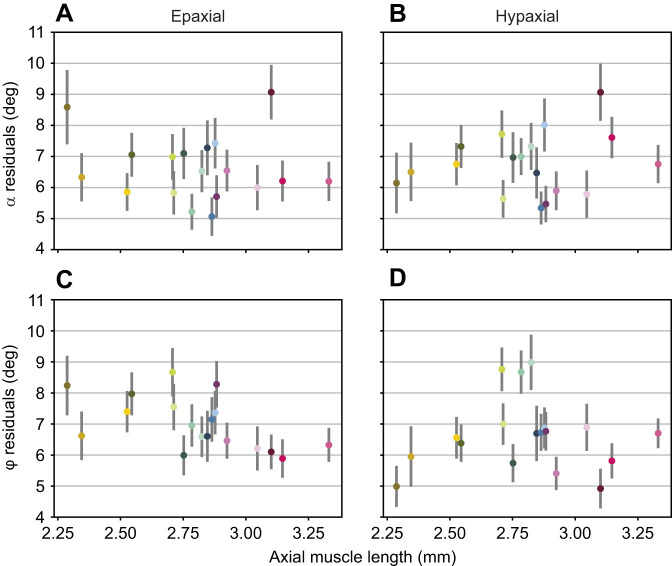
**Absolute helix fit residuals for frontal projection angle α and sagittal projection angle ϕ in the epaxial and hypaxial muscles.** (A,B) α and (C,D) ϕ in the epaxial (left) and hypaxial muscles (right), plotted against axial muscle length as proxy for fish length. Each dot represents the mean residual for all digitized muscle fibres of an individual fish, colour coded as in Fig. 1. The grey lines represent the 95% confidence interval.

**Fig. 8.**
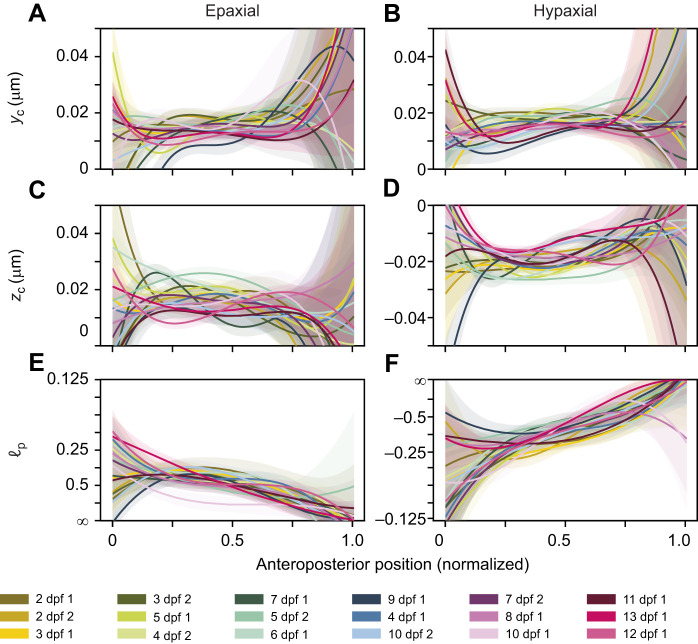
**Helix parameters for each individual fish as a function of normalized *z*-location.** Solid lines represent optimal values and shaded areas represent 95% confidence intervals. The left column shows results for the epaxial muscles, the right column for hypaxial muscles. (A,B) Dorsoventral position of the helix centre, *y*_c_. (C,D) Mediolateral position of the helix centre, *z*_c_. (E,F) The relative helix pitch (ℓ_p_) on a non-linear inverted *y*-axis, where a pitch of 0.25 indicates that four helix turns are completed within the axial muscles. The key identifies each fish's age (dpf) followed by their age-specific identification number.

In general, we found no consistent, systematic errors in frontal projection angles (see also Fig. S2). For sagittal projection angles, however, we do see systematic deviations. Typically, for the epaxial muscles, the fitted angles are higher in the first quadrant, representing the dorsolateral direction, and lower in the third quadrant, representing the ventromedial direction. In the hypaxial muscles, this pattern is mirrored relative to the horizontal septum. Positive deviations are found in the second quadrant (the dorsomedial direction) and lowest values in roughly the opposite (ventral–lateral) direction. This pattern was similar in many other individuals (Fig. S3), and reflects the fact that helices often appear ellipsoid rather than circular, with ellipsoid orientations between roughly 30 and 60 deg (in opposite directions for epaxial and hypaxial muscles; Figs 3–6).

**Fig. 9.**
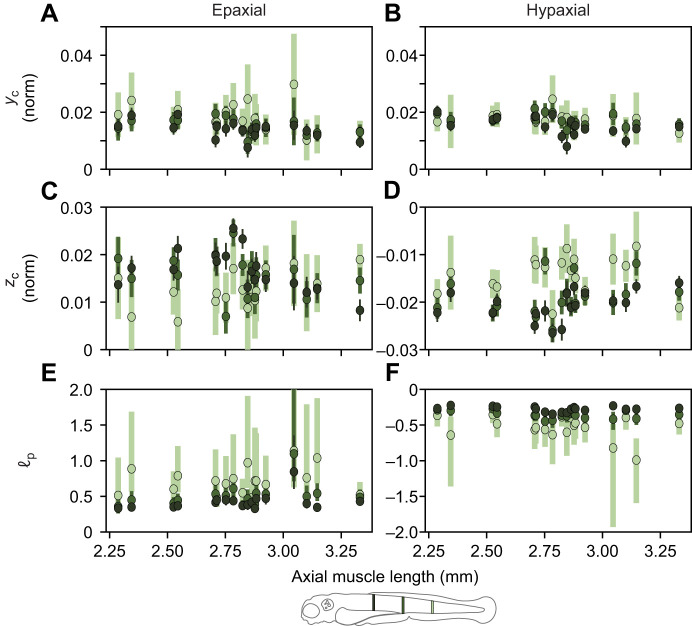
**Helix parameters as a function of fish length at three locations along the fish's anteroposterior axis.** Data points and error bars show optimal values and 95% confidence for each individual fish. Parameters for epaxial and hypaxial muscles are shown in the left and right column, respectively. (A,B) Dorsoventral position of the helix centre, *y*_c_. (C,D) Mediolateral position of the helix centre, *z*_c_. (E,F) The relative helix pitch (ℓ_p_), where a pitch of 0.25 indicates that four helix turns are completed within the body. The locations represent 0.3, 0.5 and 0.7 on the normalized anteroposterior axis (see icon at the bottom).

In the current study, we were primarily interested in analysing developmental changes in the muscle architecture. To check for changes in the quality of the fit with age, i.e. how well the pattern fits the simple helix model, we therefore compared the magnitude (absolute value) of the fit errors (residuals) across developmental stages. Fig. 7 shows the mean residuals with 95% confidence intervals as a function of axial muscle length (as a proxy for developmental stage). Both frontal (Fig. 7A,B) and sagittal projection angles (Fig. 7C,D) show considerable fit errors. However, neither the epaxial (Fig. 7A,C) nor the hypaxial part (Fig. 7B,D) reveals consistent changes with developmental stage. Thus, mismatches relative to a pattern with concentric, cylindrical helices neither increase nor decrease during development in the investigated age range. In addition to changes in the quality of the fit (residuals), we also analysed to what extent the helix parameters vary with developmental stage (Figs 8 and 9). The helix model has only three main parameters: the *y*- and *z*-coordinates of the centre of the helix (where muscle-fibre orientations are longitudinal) and the variation in projection angles α and ϕ with distance to this centre. These angles are modelled according to Eqn 12 and Eqn 13, where the scaling parameter *c* represents an effect proportional to the radial distance to the helix centre *R*. For helices with a circular cross-section, the slope *c* in these equations (and the related pitch) is the same for α and ϕ and for all directions (ζ, the angle around the cross-sectional circle of the helix) relative to the centre. To account for variation along the length of the fish, the centre coordinates as well as the slope were defined as polynomial functions of *x*. Fig. 8 shows centre coordinates and pitch as a function of the normalized *x*-axis. In the middle section, from about 0.3 to 0.7, the data show consistent behaviour with relatively narrow confidence intervals. In the most anterior and posterior parts, however, we see large variation in fitted model parameters. In these regions, the data constrain the model parameters rather poorly. In the tail region, this directly relates to diminishing pitch angles, illustrating nearly longitudinal fibre orientations at *x*-coordinates close to 1. Without a variation in orientation, the location of the helix centre is ill-defined and may end up outside the body. In the most anterior part, we see large variations in both pitch and the centre location of the helix, in combination with large confidence intervals. In this region, many other factors may play a role, such as the attachment of muscle fibres to the cranium and the presence of the pectoral girdle, causing deviations from a regular helix. In addition, in the anterior and posterior muscle portions, a relatively low number of segmented fibres is available, which reduces the effect of these regions on the model fit.

The most obvious difference between hypaxial and epaxial musculature is the sign of the slope parameter. A negative slope for hypaxial muscles corresponds to an opposite rotational direction of the helical pattern. In the middle area, where model parameters are well constrained, one can observe several additional differences between the epaxial and hypaxial musculature. On average, the hypaxial slope parameter is more extreme than the epaxial one, indicating larger changes of angle as a function of distance to the centre. The location of the centre itself also shifts along the longitudinal axis, most obviously towards the medial septum going from anterior to posterior (Fig. 8C,D). The distance to the horizontal septum (Fig. 8A,B) is relatively independent of longitudinal location.

To specifically examine variation in helix structure as a function of developmental stage, we plotted helix parameters *y_c_*, *z_c_* and pitch as a function of fish age, for three locations along the length of the fish. For the dorsoventral location of the centre (Fig. 9A,B), we see no clear dependency on fish age. For the mediolateral location (Fig. 9C,D), we see an effect of location in the fish, but also no effect of fish age. These locations are expressed in normalized distance values. As fish grow by about 50% in length over the examined period, the data reflect that growth of muscle volume may shift the location of the centre measured in absolute distance units. For helix pitch (Fig. 9E,F), no clear pattern emerges. We further examined whether helix pitch varied with axial muscle length and body location using a linear mixed-effects model with a random intercept for each fish. Observations were weighted by inverse-variance derived from the helix pitch confidence intervals. We found a small but non-significant trend for helix pitch to increase towards ∞ with fish length for both epaxial (β=−0.19±0.11 ±s.e., *t*=−1.79, *P*=0.09) and hypaxial (β=0.21±0.11, *t*=1.93, *P*=0.07) muscles. Body location did affect helix pitch, as was already clear from Fig. 8E,F, with posterior locations exhibiting higher values than anterior locations (30% versus 70%: β=0.529±0.036, *t*=14.7, *P*<0.001).

## DISCUSSION

From hatching, larval bony fish must execute rapid escape manoeuvres to survive intense predation pressure (Litvak and Leggett, 1992). These fast movements rely on the mechanical power produced by the axial muscles. In juvenile and adult bony fish, muscle fibres follow pseudo-helical trajectories (Mos and Van der Stelt, 1982; van Leeuwen et al., 2008). To explore early muscle architecture development, we quantified fibre orientation in larval zebrafish (*Danio rerio*) from 2 to 13 dpf. Using confocal fluorescence microscopy and automated segmentation and quantification, we obtained high-resolution measurements along the entire axial musculature, while minimizing distortion artifacts. This enabled us to fit the proposed helical pattern to both the epaxial and hypaxial muscles of individual fish.

Our data show that the helical fibre pattern, described in detail for 4 dpf larvae by van Meer et al. (2025), is already present immediately after hatching at 2 dpf and remains largely unchanged until 13 dpf. In addition, a scan of a pre-hatching larva at 1 dpf reveals muscle fibres that are already oriented at angles deviating from the body axis. These findings contrast with those of Dou et al. (2008), who, using low-angle X-ray diffraction, observed mostly longitudinal fibres at 5 and 7 dpf. In contrast, we observed clear helical patterns as early as 2 dpf, with frontal and sagittal projection angles exceeding 30 deg anteriorly. This challenges the findings of van Raamsdonk et al. (1974), who described helical trajectories emerging only by 6–7 dpf.

Epaxial and hypaxial muscle fibre patterns are mirrored across the horizontal septum: hypaxial fibres rotate clockwise along the body axis, epaxial fibres counter-clockwise. In the helix model, this corresponds to a sign inversion. Anteriorly and posteriorly, helical parameters were less well defined. In the tail, this is due to the limited number of data points combined with fibres running longitudinally. In the anterior region, the fitted parameters were also more variable and poorly constrained, probably reflecting the broader range of projection angles seen in this area – presumably influenced by head-related specializations as well as potential sampling biases. Interestingly, we did not observe pronounced epaxial–hypaxial asymmetries in the anterior region, despite differences in their transverse cross-sectional areas. Instead, fibre angles appear symmetrically defined relative to the notochord (see also Figs 3 and 4), supporting the use of the notochord as a central reference in the helix model. When considering the functional consequences of fibre orientation for generating body undulations, the flexural properties of the anterior notochord should also be taken into account.

The early presence of a helical pattern suggests it is largely innate rather than shaped by swimming feedback. Although larval muscle activation begins pre-hatching, early movements differ significantly from post-hatching swimming. Effective escape manoeuvres are essential for survival immediately after hatching, and by around 5 dpf, larvae must also be capable of precise prey-capture movements. The early appearance of the helical pattern may therefore reflect its high survival value, supporting the evolutionary acquisition of a complex helical muscle fibre arrangement. The early presence of the helical pattern does not exclude a role for swimming activity in further shaping the muscle architecture; muscle use may still refine fibre orientation, as seen in *nicb107* mutant zebrafish lacking muscle activity (van der Meulen et al., 2005) In our data, we found no clear structural changes between 2 and 13 dpf, but clearly the structure at that age is still quite different from that in juveniles or adults. For example, adults show the presence of smaller, additional helices, and in juveniles, the main helix structure has been described as pseudo-helical, with deviations in frontal projection angles near the median plane.

The helical pattern can contribute to effective swimming in several different ways. As suggested by Alexander (1969) it may contribute to minimizing strain variation across (part of) the axial musculature. For fibres running in the longitudinal direction, the strain increases from near-zero at the median plane to a maximum at the most lateral locations. Within a transversal cross-section, most longitudinal fibres would therefore operate outside of their optimal strain regime. Alexander (1969) correctly noted that the helical pattern can equalize strains by increasing the angle relative to a longitudinal direction for more lateral locations. However, medial to the centre of the helices, the increasing ϕ angles do not increase the strain relative to that in the more laterally located helix centre. In fact, a helix shape predicts fibres running parallel to the median plane, resulting in zero strain at the height of the helix centres. van der Stelt (1968) instead suggested that, to be able to shorten and thus contribute to power generation, medial fibres should make significant angles to the median plane, resulting in shear deformation during contraction. Mos and Van der Stelt (1982) and van Leeuwen et al. (2008) found evidence for high azimuth angles near the median plane at the level of the helix centre, and van Leeuwen et al. (2008) showed that strain variation can indeed be minimized across a transverse muscle slice by the combination of an ‘added’ shear deformation and the pseudo-helical pattern with high azimuth angles near the median plane. It remains unclear to what extent the (pseudo-)helical pattern can provide a general solution for all swimming modes as its effect on minimizing strain variation may vary with body curvature. Additionally, it remains unclear to what extent this pattern can provide the required dorsoventral strain gradient during suction feeding (Jimenez et al., 2021).

We did not find clear indications for the pseudo-helical arrangement in our data. It would show up as high frontal projection angles close to the median plane at the dorsoventral level of the helical centres. This was not obvious in our data, at any of the developmental stages. For example, we do not see higher fit residuals on the medial side of the helix centres in Fig. 6 and Fig. S2. In fact, the residuals in frontal projection angle show less structure than those in sagittal projection angle. The fact that we did not find elevated frontal projection angles relative to the perfect helical pattern does not rule out that shear deformation plays an important role in minimizing strain variation. Shear is still possible, even for nearly longitudinal fibre directions if the shape of myosepts, on which the fibres are inserted, allows for shear, e.g. by changing the angle of the myosepts relative to the median plane upon contraction. One possible reason why previous studies that used microscopic slides of fixed tissues did find elevated frontal projection angles, and thus a pseudo-helical pattern, might be that tissue fixation caused (partial) muscle-fibre contractions leading to fixation in the sheared deformation state. We worked with living anaesthetized larvae and recently euthanized larvae, so we presumably measured orientations in the rest state. An alternative explanation is that we did not see a clear pseudo-helical pattern because the shear component develops only later in development, possibly in an activity-dependent way.

In addition to minimizing strain variation, the helical pattern may also play a role in uncoupling the effects of muscle contractions along the length of the larval body. The generation of bending moments to propel the fish requires a travelling wave of muscle contractions from anterior to posterior along the body. Although presumably about a factor of two faster than the wave of curvatures (Voesenek et al., 2020), anterior contractions are out of phase with posterior contractions. This can only be achieved if contraction of anterior fibres is uncoupled from length changes in posterior fibres. For longitudinally arranged fibres, anterior contractions would be partly dissipated by stretching of more posterior parts. By placing all muscle fibres at an angle relative to the longitudinal axis, forces are directed towards the skin or the medial axis (notochord), thus reducing posterior stretching. In this view, the pitch of the helix determines the length over which forces are redirected, and the length over which forces are transmitted posteriorly before ‘connecting’ to either skin or median plane. We found pitches in the anterior part of the muscles that were about a quarter of the fish length. This may add to localizing the effects of muscle contractions and thus supports the generation of a wave of bending moments. In addition, the myosepts may help to prevent force unbalances between neighbouring myomeres and to transmit excess forces towards the skin and median plane (van Leeuwen, 1999).

Our data clearly show how the helical pattern varies along the length of the fish. Short pitches are found anteriorly, corresponding to steep increases of projection angle with radial distance to the helix centre. More posteriorly, the angle decreases and pitch increases. The helix pitches were relatively shorter for the hypaxial muscles than for epaxial muscles, corresponding to higher projection angles hypaxially. These patterns were previously described for 4 dpf fish (van Meer et al., 2025). Here, we show that this is consistent across different developmental stages. How this change in pitch from anterior to posterior affects, for example, the minimization of the variation in strain is not directly obvious. In addition to the pitch, the shear deformation could also vary with the anteroposterior location. To gain insight in these different effects, one could use the bending beam model to quantify strains for different projection angles and for different amounts of shear (van Leeuwen et al., 2008). A more comprehensive approach may also take hydrodynamics and activations during different phases of the curvature wave into account. The latter approach boils down to a full, forwards-dynamic model, which is, however, restricted by a lack of knowledge on, for example, mechanical properties of connective and supporting tissues. The fact that we found similar patterns for all developmental stages suggests that limited changes in fibre direction are required to optimize swimming efficacy with age.

This is the first study to use a geometric model to describe the muscle-fibre architecture in larval fish. We used a helix model to describe the muscle-fibre arrangement with three parameters that vary with anteroposterior location *x* along the notochord: (1) the dorsoventral (*y*) and (2) mediolateral (*z*) positions of the helix centre, and (3) the helix pitch (ℓ_p_). We made separate fits for the epaxial and hypaxial muscles. For the fit, we computed the least-squares-error sum of the frontal (α) and sagittal (ϕ) projection angles. These angles specify the orientation of a muscle fibre as projections on two orthogonal planes. In this way, we could also examine whether deviations from the model occur in a specific orientation only. We found deviations especially in the sagittal projection angles (Fig. 6; Fig. S3). For the epaxial muscles, measured sagittal projection angles were too high in the dorsolateral direction and in the ventromedial direction relative to the helix centre. For the hypaxial muscles, this pattern was mirrored around the horizontal septum. These errors are in line with an ellipsoid rather than circular cross-sectional shape of the helices, where the orientation is such that the ellipsoid orientation matches the orientation of the bulk of the musculature. In an extended model description, we could add an ellipsoid shape of the helix, with an arbitrary rotation relative to the longitudinal axis. Finding the best model description would also require investigating additional variation, such as a variable pitch with distance to the helix centre. Finding the model with the best goodness of fit requires comparing, for example, the Akaike information criterion for all different model complexities, which is beyond the scope of the present study.

In addition to some systematic deviations, we also found a high degree of random deviations. These random deviations may partly result from shortcomings in fibre segmentations. Our analysis was aimed at segmenting individual fibres, based on differences in fluorescence brightness between adjacent fibres. Imperfect segmentations might have resulted in erroneous fibre shapes, and thus in deviations in estimated fibre orientation. Orientation estimates may also have been compromised by a changing shape of cross-sections over the length of a fibre. The local shape is partly determined by the number and location of surrounding fibres, which for helical patterns may change over the length of a fibre, adding noise to the obtained projection angles. Such variation is partly inherent to the analysis procedure, and partly reflects actual variation in orientation. Interestingly, we did not find a reduction in residuals with age, making it unlikely that either noise or systematic variation changed with age. For more complex models, that dive deeper into the details of the helical structure, it might be worthwhile to increase the number of valid measurements per fish and at the same time reduce measurement noise.

### Conclusion

We modelled muscle-fibre orientation in larval zebrafish as a set of concentric helices in which the helix pitch does not vary with the distance *R* to the centre of the helices. Measured muscle-fibre orientation and model fits suggest that the hypothesized helical muscle-fibre arrangement is present from the moment a zebrafish larva hatches, and remains largely unaltered up to an age of 13 dpf. Moreover, this pattern may already be present pre-hatching as muscle fibres in a 1 dpf larva deviate from a longitudinal orientation. Neither goodness-of-fit nor model parameters change substantially with age. Our study quantitatively compared muscle-fibre orientation with a helical model over the entire axial muscle volume in developing larval fish, providing new insights into early life muscle adaptations.

## Supplementary Material

10.1242/jexbio.250905_sup1 Supplementary information
