# Supplementary material for: Axial muscle-fibre orientation in developing larval zebrafish
Source: J Exp Biol. 2026 Jan 2;229(1):jeb250905. doi: 10.1242/jeb.250905 (PMC12813669; doi:10.1242/jeb.250905)
Supplement: Supplementary information [file jexbio-229-250905-s1.pdf]

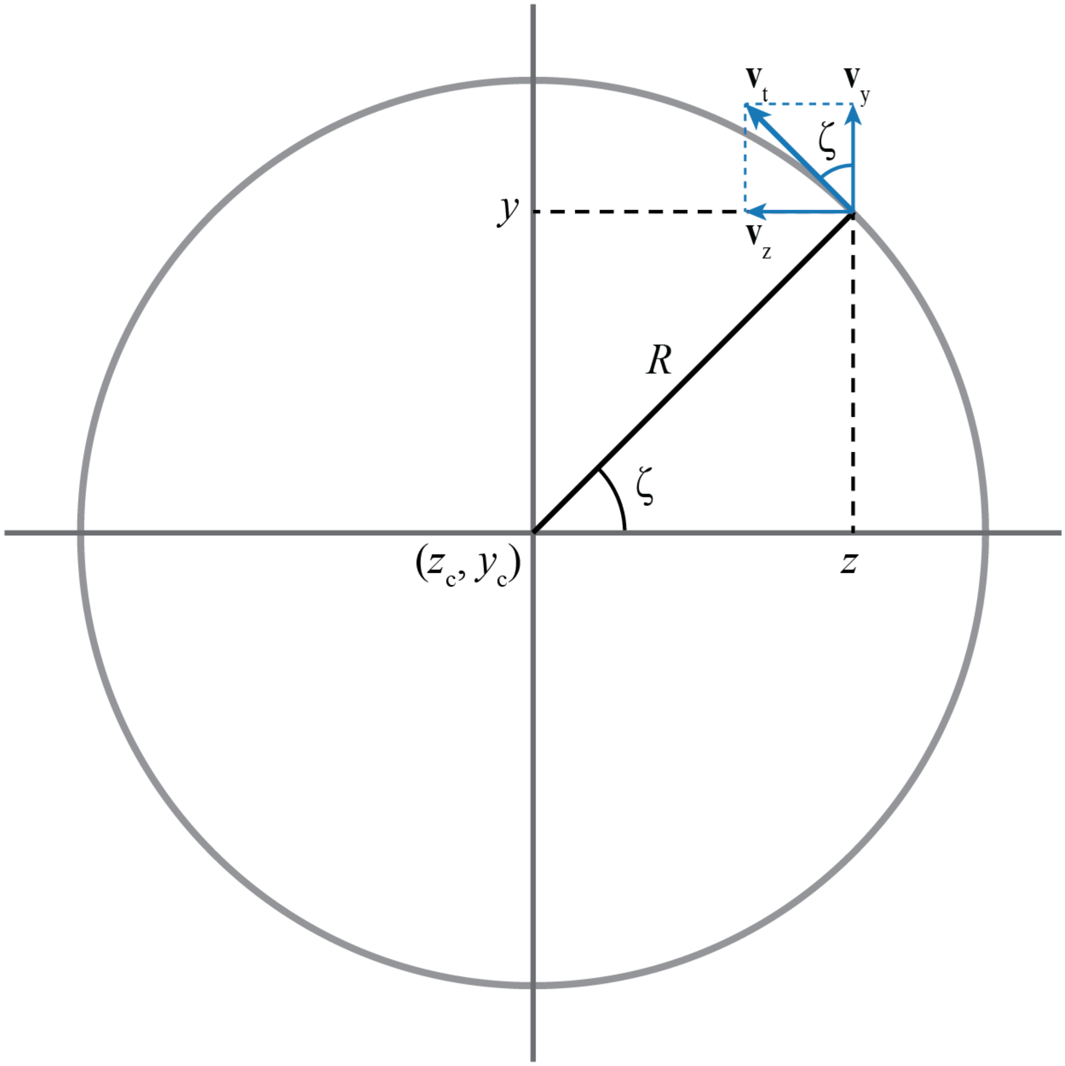

Fig. S1. Visualization of model equations 1-5. For a point on a helix (x, y, z) and helix centre (z_c, y_c), R is the radial distance to the helix centre, ζ is the angle around the cross-sectional circle of the helix, and v = (v_x, v_y , v_z) is the tangent to the helix, with its projection in the transverse plane v_t.

Journal of Experimental Biology • Supplementary information

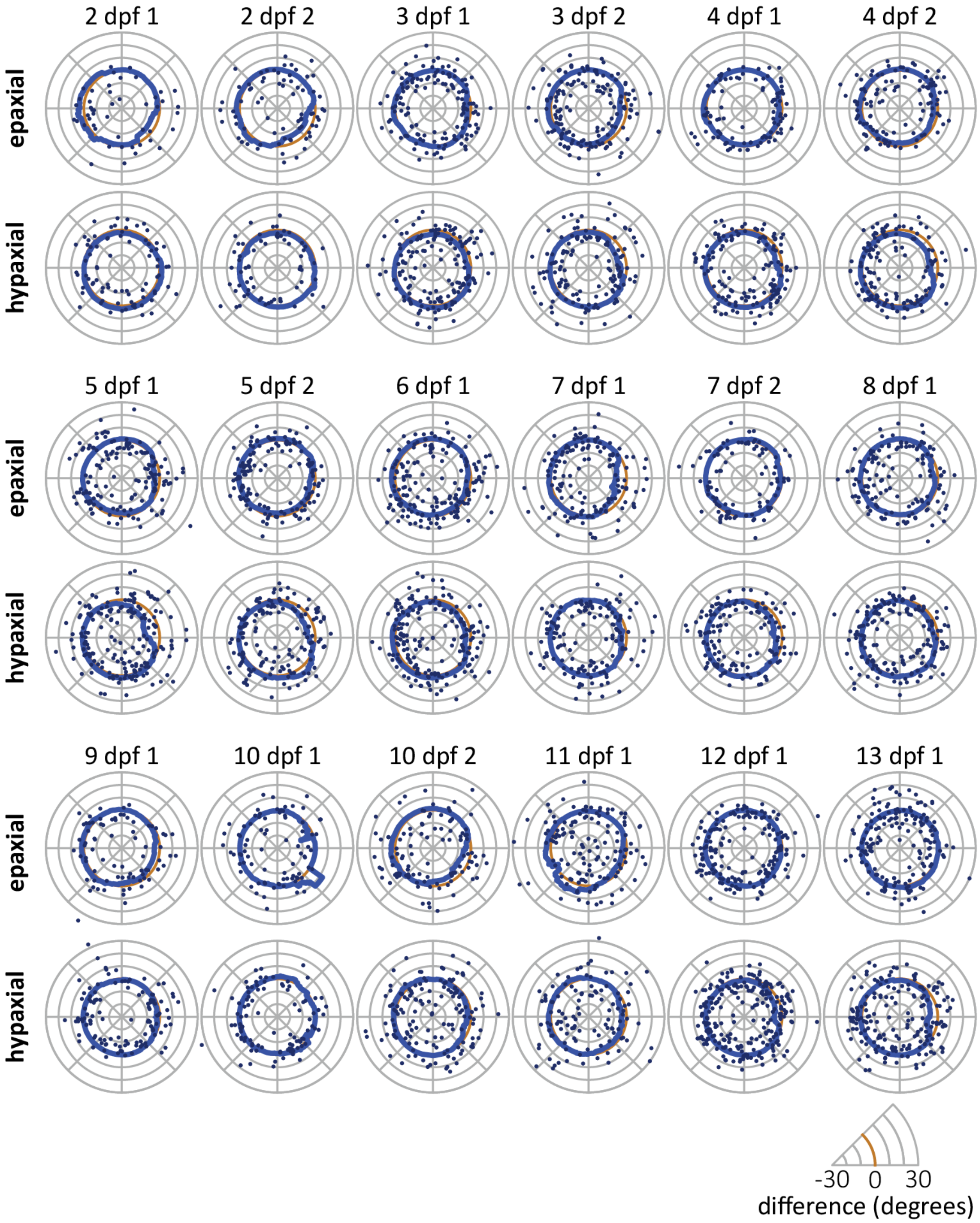

Fig. S2. Polar plots with directional differences in frontal projection angle α around the helix centre, as determined by the model for each individual. The plots show the muscle segment between 0.4 and 0.8 on the normalized anteroposterior axis. The solid blue line represents a moving average with a 90-degree window. Each fish is depicted with both epaxial and hypaxial muscles, labeled above each plot pair. Labels include each fish's age (x dpf) followed by their age-specific identification number.

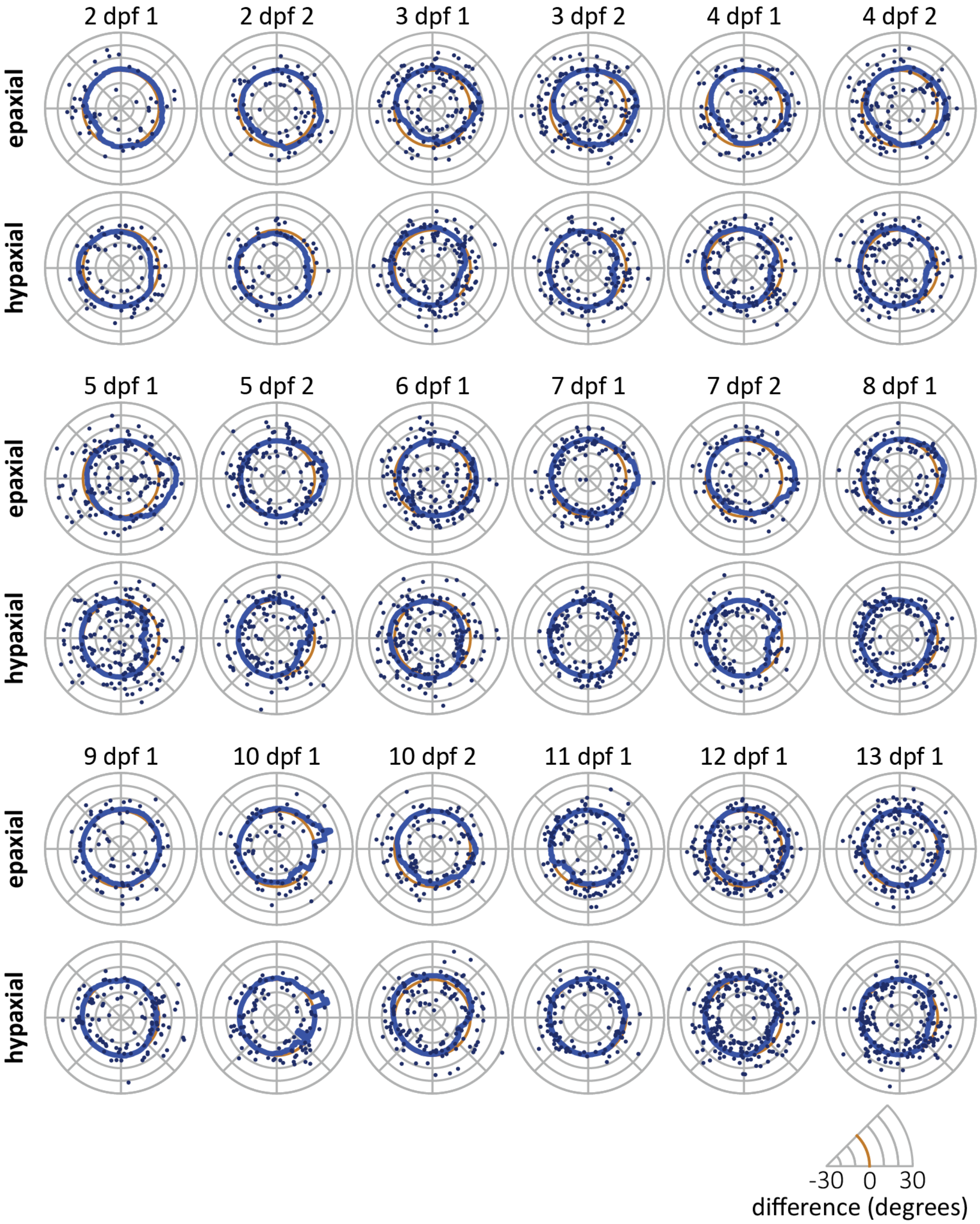

Fig. S3. Polar plots with directional differences in sagittal projection angle φ around the helix centre, as determined by the model for each individual. The plots show the muscle segment between 0.4 and 0.8 on the normalized anteroposterior axis. The solid blue line represents a moving average with a 90-degree window. Each fish is depicted with both epaxial and hypaxial muscles, labeled above each plot pair. Labels include each fish's age (x dpf) followed by their age-specific identification number.

Journal of Experimental Biology • Supplementary information
